# Prevalence, Predictors, and Outcomes of Pulmonary Hypertension in Patients with Lupus Nephritis

**DOI:** 10.3390/medicina60060988

**Published:** 2024-06-17

**Authors:** Sixiu Chen, Junhan Guo, Xiamin Huang, Wei He, Xueqing Yu, Xi Xia, Wei Chen

**Affiliations:** 1 Department of Nephrology, The First Affiliated Hospital, Sun Yat-sen University, NHC Key Laboratory of Nephrology (Sun Yat-sen University), Guangdong Provincial Key Laboratory of Nephrology, Guangzhou 510080, China; chensx53@mail.sysu.edu.cn (S.C.); guojunhan1992@163.com (J.G.); minxiagcp@163.com (X.H.); yuxq@mail.sysu.edu.cn (X.Y.); 2 Department of Ultrasound, The First Affiliated Hospital, Sun Yat-sen University, Guangzhou 510080, China; hewei49@mail.sysu.edu.cn

**Keywords:** pulmonary hypertension, lupus nephritis, mortality, kidney failure, renal dialysis

## Abstract

*Background and Objectives:* This study aimed to assess the prevalence, predictors, and outcomes of pulmonary hypertension (PH) in patients with lupus nephritis (LN). *Materials and Methods:* Baseline characteristics and clinical outcomes of 387 patients with LN were retrospectively collected from 2007 to 2017. PH was defined as pulmonary artery systolic pressure ≥40 mmHg assessed by resting transthoracic echocardiography. The primary endpoint was all-cause mortality. The secondary endpoint was renal events, defined as the doubling of baseline serum creatinine or end-stage renal disease. Associations between PH and outcomes were analyzed by Cox regression models. *Results:* A total of 15.3% (59/387) of patients with LN were diagnosed with PH, and the prevalence of PH was higher for patients with an estimated glomerular filtration rate (eGFR) < 30 mL/min/1.73 m^2^ compared to those with an eGFR ≥ 30 mL/min/1.73 m^2^ (31.5% vs. 12.6%). Higher mean arterial pressure, lower hemoglobin, and lower triglyceride levels were associated with greater odds of having PH. After adjusting for relevant confounding variables, PH was independently associated with a higher risk for death (HR: 2.01; 95% CI: 1.01–4.00; *p* = 0.047) and renal events (HR: 2.07; 95% CI: 1.04–4.12; *p* = 0.039). *Conclusions:* PH is an independent risk factor for all-cause mortality and adverse renal outcomes in patients with LN.

## 1. Introduction

Pulmonary hypertension (PH) is characterized by a mean pulmonary artery pressure exceeding 20 mmHg and is commonly seen in advanced diseases [1,2]. Connective tissue disease (CTD) is the second leading cause of PH after the idiopathic form and has a poorer survival rate compared to idiopathic PH, with an overall risk factor of 1.59 [3,4]. Although systemic sclerosis represents the main CTD associated with PH in Europe and the United States, systemic lupus erythematosus (SLE)-associated PH is more common in Asia, with a prevalence of 35–60% [5,6,7]. Moreover, the long-term prognosis of SLE-associated PH is poor, with a 5-year survival rate of 68–72.9% [8,9].

SLE presents with various clinical and immunologic manifestations, with lupus nephritis (LN) as the primary driver of morbidity and mortality [10]. It has been shown that patients with chronic kidney disease (CKD) and PH have a significantly higher all-cause mortality [11]. However, there is a lack of comprehensive understanding regarding the prevalence and risk factors of PH in patients with LN, as well as the impact of concurrent LN and PH on mortality and renal outcomes.

The gold standard for diagnosing PH is right heart catheterization (RHC); however, it is invasive and unsuitable for screening in large cohorts [6]. Therefore, Doppler echocardiography has been introduced and noninvasive transthoracic echocardiography has been validated as an acceptable screening tool for PH in patients with CTD [12]. Herein, we investigated the prevalence, predictors, and outcomes of PH assessed through echocardiography in a large cohort of LN patients.

## 2. Materials and Methods

### 2.1. Study Design

From January 2007 to March 2017, registered patients with LN who were older than 14 years old and met the revised American College of Rheumatology (ACR) criteria (1997) were retrospectively included in the lupus nephritis database (http://ln.medidata.cn, accessed on 1 May 2017) of the Department of Nephrology, the First Affiliated Hospital of Sun Yat-sen University [13]. The study was approved by the Ethics Committee of the First Affiliated Hospital of Sun Yat-sen University. Written informed consent was obtained from each participant.

### 2.2. Patients

The exclusion criteria were as follows: (1) end-stage kidney disease (ESKD) on admission; (2) presence of other types of PH caused by chronic pulmonary disease or heart disease; (3) presence of other CTDs that can cause PH; (4) absence of echocardiography data; and (5) absence of follow-up data.

### 2.3. PH Definition and Covariates

#### 2.3.1. PH Definition and Pulmonary Artery Systolic Pressure (PASP) Equation

An echocardiographic examination was performed by experienced echocardiographers on patients in the resting position. PH was defined as a PASP ≥ 40 mmHg [14] and PASP was calculated using the modified Bernoulli equation:

PASP = 4 × (tricuspid systolic jet) 2 + right atrial pressure [14,15].

#### 2.3.2. SLE Disease Activity

SLE disease activity was assessed according to the revised Systemic Lupus Erythematosus Disease Activity Index (SLEDAI) scores [16]. Systemic manifestations (vasculitis, arthritis, myositis, rash, oral ulceration, pleuritis, pericarditis, fever, leukocytopenia, and thrombocytopenia) were assessed using the SLEDAI, and all occurrences were classified according to SLEDAI definitions. The SLEDAI scores were calculated retrospectively by reviewing patients’ records.

#### 2.3.3. Estimated Glomerular Filtration Rate (eGFR)

The eGFR was calculated using the 4-variable Modification of Diet in Renal Disease (MDRD) formula as follows:

eGFR (mL/min/1.73 m^2^) = 186 × serum creatinine (mg/dL) − 1.154 × age − 0.203 × (0.742 if female) [17].

For adolescent patients aged 14–18 years, the Schwartz formula was used:

eGFR (mL/min/1.73 m^2^) = k × height (m)/serum creatinine (mg/dL) (for girls and boys < 13 yr: k = 48.6; for boys > 13 yr: k = 61.9) [18].

#### 2.3.4. Mean Arterial Pressure

Mean arterial pressure = (systolic blood pressure + 2 × diastolic blood pressure)/3.

#### 2.3.5. Other Covariates

All patients undergoing renal biopsy were reviewed and reclassified according to the 2003 Classification System of the International Society of Nephrology/Renal Pathology Society [19], as well as the activity index and chronic index scores. The duration of SLE and LN was defined as the time from onset to admission.

Urine protein excretion was calculated from the 24 h urine collection. The reference values for C3 and C4 were 0.79–1.17g/L and 0.17–0.31g/L, respectively. Low C3 and C4 were defined as less than 0.79 g/L and 0.17 g/L, respectively. The investigators received training on diagnostic confirmation, disease activity evaluation, data input, and data quality control. Demographic data (age, sex, and disease duration), echocardiography findings, and radiological, clinical, laboratory, and autoantibody data were obtained.

### 2.4. Endpoints

The primary endpoint was all-cause mortality. The secondary endpoint was renal events, defined as doubling of baseline serum creatinine or end-stage renal disease (maintenance dialysis, renal transplantation, or eGFR < 15 mL/min/1.73 m^2^). All patients were followed up until 2 September 2018, and the follow-up was censored at the end of the follow-up time, loss to follow-up, or death, whichever occurred first.

### 2.5. Analysis

The data were expressed as means ± standard deviations, medians (interquartile ranges), and frequencies (%) as appropriate. The differences between the two groups were assessed by *t*-test or Mann–Whitney U test for the normally and non-normally distributed continuous variables, respectively, and by the chi-square test for categorical variables. The odds ratios (ORs) for PH were calculated based on multivariate logistic regression analyses adjusted for potential confounding variables that were significant in univariate logistic regression analyses (*p* < 0.1) and using a stepwise conditional method (probability for stepwise: entry, 0.1; removal, 0.1) [20]. Kaplan–Meier survival curves were plotted to calculate the cumulative event-free survival rates and were analyzed by the log-rank test. Figures of log minus log were performed to assess the assumption of proportional hazard, and these checks were satisfied. Cox regression models were used to evaluate the relationship between PH with all-cause mortality and renal events, initially without adjustment and subsequently adjusting for several groups of covariates. The multivariate Cox regression model was constructed using eligible covariates that were important for clinical concerns [21]. *p* values < 0.05 were considered statistically significant. SPSS version 20.0 (SPSS Inc., Chicago, IL, USA) was used for statistical analysis.

### 2.6. Patient and Public Involvement

There was no involvement from patients or members of the public in the design, conduct, reporting, or dissemination plans of the research.

## 3. Results

### 3.1. Prevalence and Baseline Characteristics

**Figure 1:**
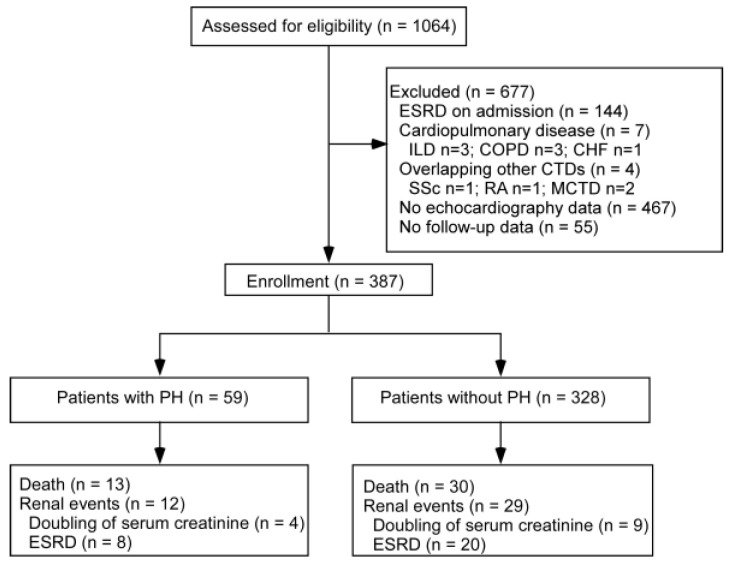
Enrollment flow chart and outcomes of patients with lupus nephritis. CHF, chronic heart failure; COPD, chronic obstructive pulmonary disease; CTD, connective tissue disease; ESRD, end-stage renal disease; ILD, interstitial lung disease; MCTD, mixed connective tissue disease; PH, pulmonary hypertension; RA; rheumatoid arthritis; SSc, systemic sclerosis.

In total, 378 patients were enrolled in this study, and PH was present in 15.3% (59/378) of patients in this LN cohort (Figure 1). The prevalence of PH increased when the eGFR was less than 30 mL/min/1.73 m^2^. For patients with CKD stages 1, 2, 3, and 4, the prevalence of PH was found to be 11.0%, 14.8%, 13.1%, and 31.5%, respectively. The baseline demographic and clinical characteristics of the study population are summarized in Table 1. Patients with PH had significantly higher mean arterial pressure and were more likely to have pleuritis, higher serum creatinine levels, and higher C-reactive protein levels. Furthermore, patients with PH had significantly lower levels of hemoglobin and fewer triglycerides (TGs) than those without PH. LN patients who had echocardiography performed were older and had higher mean arterial pressure, lower hemoglobin, higher serum creatinine, higher 24 h proteinuria, and higher SLEDAI.

### 3.2. Risk Factors for PH in Patients with Lupus Nephritis

Logistic regression analysis was performed to determine the risk factors of PH. The multivariate analysis identified that higher mean arterial pressure (OR, 1.05; 95% confidence interval [CI], 1.03–1.08; *p* < 0.001), lower hemoglobin (OR, 0.98; 95% CI, 0.97–1.00; *p* = 0.014), and lower TG were (OR, 0.71; 95% CI, 0.52–0.98; *p* = 0.038) independently associated with higher odds of PH (Table 2). The factors involved in the univariate analysis associated with PH were listed in Supplementary Table S1.

### 3.3. Association between PH and Clinical Outcomes

**Figure 2:**
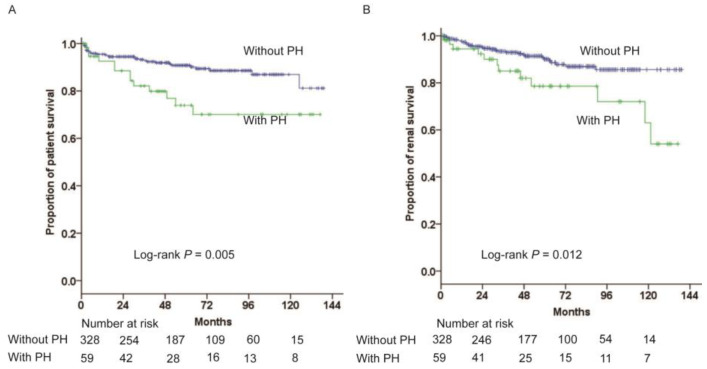
Kaplan–Meier survival curve analysis for all-cause mortality (**A**) and renal events (**B**) for those patients with and without pulmonary hypertension. PH; pulmonary hypertension.

During the median follow-up of 52 months (interquartile range: 25–85 months), 43 patients died and 41 patients had renal events. LN patients with PH showed 1-, 3-, and 5-year survival rates of 93%, 82%, and 73%, compared to rates of 95%, 93%, and 91% for patients without PH. Consequently, patients with PH exhibited significantly higher all-cause mortality rates (log-rank *p* = 0.005, Figure 2A). In addition, the risk of renal events increased significantly for the patients with PH when compared with those without PH (log-rank *p* = 0.012, Figure 2B). PH was independently associated with higher risks for death (hazard ratio, 2.01; 95% confidence interval, 1.01–4.00; *p* = 0.047) and renal events (hazard ratio, 2.07; 95% confidence interval, 1.04–4.12; *p* = 0.039), after adjusting for age, gender, serum creatinine, serum albumin, and 24 h proteinuria (Table 3).

## 4. Discussion

To the best of our knowledge, this is the largest study so far to assess the long-term prognosis of LN patients with PH. In this retrospective study, we revealed that the prevalence of PH was 15.3% in a Chinese LN cohort and the prevalence of PH increased to 31.5% when eGFR < 30 mL/min/1.73 m^2^. We have identified that higher mean arterial pressure, lower hemoglobin levels, and lower TG content were independently associated with higher odds of PH in patients with LN. Moreover, for the first time, our data indicated that PH was an independent risk factor for mortality and adverse renal events in LN patients.

The prevalence of PH previously reported for SLE patients was 2.06–3.8% in large national cohort studies in China [7,22,23], 4.2% in a prospective cross-sectional study in the United Kingdom [24], and 2.99% in a prospective single-center study in Spain [25]. Noteworthy, the overall prevalence of PH in our LN cohort was 15.3%, which is significantly higher than the prevalence reported in SLE patients. It is unclear whether LN increases the risk of developing PH in patients. However, the Chinese SLE Treatment and Research Group (CSTAR) found that the prevalence of LN was higher in SLE patients with PH than in non-PH patients [22], and the prevalence of PH was reported to be 21–27% in patients with non-dialysis-dependent CKD [11,26,27,28]. Previous studies and our findings suggest that SLE patients with LN may be at a higher risk of having PH than those without LN. However, whether this elevated risk is caused by LN or by comorbid CKD still requires further study. Future large epidemiologic studies could help clarify this issue by examining the prevalence of PH in SLE patients with or without LN and comparing the prevalence of PH in patients with LN to those with CKD of other etiologies.

Our study has identified that the risk of PH increased with higher mean arterial pressure, lower hemoglobin, and lower TG. In agreement with our study, systemic hypertension was a risk factor for PH development in the SLE population [29], and the presence of anemia was significantly associated with PH in a CKD study [11]. The TG levels are typically elevated in most patients with SLE and LN, contributing to systemic inflammation and oxidative stress, which exacerbate renal damage in LN [30,31]. However, in our study, we observed lower TG levels as risk factors for PH. We hypothesize that this finding may be explained by the excessive right ventricular volumes and pressures induced by PH. Some studies have suggested that TG levels are reduced in patients with PH because the synthesis of TG depends on the liver, which is commonly the first organ to be affected by right ventricular failure in patients with PH [32]. A cohort study of 442 unscreened patients with congestive heart failure found a correlation between right ventricular end-diastolic diameter and decreased levels of circulating lipids, including TG, HDL, and LDL [33]. Our results suggest that lower TG levels may serve as a new biomarker for the development of PH in patients with LN, but further studies are needed to validate this finding.

The 5-year survival rate was 73% in LN patients with PH in our study, which is similar to the 5-year survival rate in SLE-associated PH, that is 68–83.9% [7,8,9,29,34]. Consistent with previous studies that suggested PH was associated with a higher risk of mortality in the general population and in those with CKD, ESRD, or SLE [35,36,37,38,39], our data showed that PH was an independent predictor of mortality for patients with LN. Furthermore, cardiovascular events ranked as the second leading cause of death, while infections were the primary cause, aligning with observations in SLE patients [40]. Nonetheless, owing to the limited number of cardiovascular mortality cases in our sample, we could not definitively ascertain the association between PH and cardiovascular mortality. Notably, our findings revealed that PH independently predicted adverse renal outcomes in patients with LN. The relationship between PH and renal prognosis in CKD remains debatable. While one study demonstrated that PH increased the risk of kidney failure among elderly Medicare beneficiaries with non-dialysis CKD [39], another study, like the Chronic Renal Insufficiency Cohort (CRIC) Study, did not identify PH as an independent risk factor for renal events [11]. Discrepancies across these studies can be attributed to variations in the underlying kidney diseases, study populations, and definitions of PH. Future investigations are essential to elucidate the implications of PH in kidney diseases, particularly among LN patients.

## 5. Limitations

Some limitations should be addressed. First, we excluded patients who lacked echocardiographic data, which may lead to inclusion bias, and the results may lack generalization to other populations and cohorts. Second, RHC data and other important echocardiogram variables such as right chamber heart dilatation or inversion of the interventricular septum towards the left ventricle were not available, and echocardiograms may overestimate PH. Third, pericardial effusion was very common in LN patients due to lupus and hypoproteinemia, and the differential diagnosis for pericardial effusion in these patients sometimes may be difficult in clinical practice. So, the diagnosis of pericarditis may be inaccurate, and the prevalence of pericarditis was overestimated. Fourth, most of the LN patients had only mild PH and received no PH target medications in this cohort; thus, the effect of PH target medications needs to be further studied.

## 6. Conclusions

In conclusion, our study highlighted the critical need for vigilant screening for PH in LN patients, particularly those with reduced eGFR, to facilitate early intervention and improve clinical outcomes. Further research should focus on elucidating the mechanisms linking PH and LN and on developing targeted strategies for managing this high-risk population.

## Figures and Tables

**Table 1:** Baseline characteristics of lupus nephritis patients with and without pulmonary hypertension.

Variables	Total (*n* = 387)	With PH (*n* = 59)	Without PH (*n* = 328)	*p* Value ^a^
Age(y)	30 (21, 40)	34 (23, 39)	30 (20, 40)	0.190
Female, *n* (%)	322 (83.2)	47 (79.7)	275 (83.8)	0.450
Lupus duration on admission (months)	6 (2, 30)	13 (2, 49)	6 (2, 25)	0.128
Lupus nephritis duration on admission (months)	3 (1, 19)	5 (1, 25)	3 (1, 18)	0.488
Mean arterial pressure (mmHg)	99 (88, 111)	109 (101, 119)	97 (87, 109)	<0.001
Fever, *n* (%)	105 (27.1)	17 (28.8)	88 (26.8)	0.752
Rash, *n* (%)	156 (40.3)	21 (35.6)	135 (41.2)	0.473
Ulceration, *n* (%)	17 (4.4)	3 (5.1)	14 (4.3)	0.732
Arthritis/Myositis, *n* (%)	161 (41.6)	25 (42.4)	136 (41.5)	0.887
Pleuritis, *n* (%)	128 (33.1)	29 (49.2)	99 (30.2)	0.006
Pericarditis, *n* (%)	211 (54.5)	35 (59.3)	176 (53.7)	0.479
Vasculitis, *n* (%)	5 (1.3)	0 (0)	5 (1.5)	1.000
Leukocytopenia, *n* (%)	90 (23.3)	16 (27.1)	74 (22.6)	0.503
Thrombocytopenia, *n* (%)	34 (8.8)	5 (8.5)	29 (8.8)	1.000
Hemoglobin (g/L)	98 (81, 115)	87 (75, 103)	99 (82, 116)	0.001
24 h proteinuria (g)	2.6 (1.0, 5.3)	2.7 (1.0, 5.5)	2.5 (1.0, 5.3)	0.895
Blood urea nitrogen (mmol/L)	8.2 (5.3, 13.2)	11.0 (6.0, 16.9)	7.9 (5.1, 12.7)	0.006
Serum creatinine (umol/L)	90 (64, 144)	106 (68, 228)	86 (62, 136)	0.007
eGFR (mL/min/1.73 m^2^)	71.3 (42.1, 109.4)	58.2 (25.8, 93.6)	74.0 (44.5, 111.7)	0.004
Serum uric acid (μmol/L)	419 (326, 543)	480 (333, 583)	413 (326, 533)	0.138
Serum albumin (g/L)	26 (21, 31)	26 (21, 32)	26 (21, 31)	0.835
Total cholesterol (mmol/L)	5.6 (4.3, 7.0)	5.0 (4.0, 6.5)	5.7 (4.4, 7.2)	0.069
Triglycerides (mmol/L)	2.2 (1.5, 3.0)	1.8 (1.2, 2.5)	2.2 (1.5, 3.1)	0.028
HDL-C (mmol/L)	1.0 (0.7, 1.3)	0.9 (0.7, 1.1)	0.99 (0.72, 1.34)	0.169
LDL-C (mmol/L)	3.3 (2.6, 4.5)	3.1 (2.5, 4.1)	3.4 (2.6, 4.5)	0.178
C3 (g/L)	0.41 (0.26, 0.61)	0.36 (0.25, 0.59)	0.42 (0.27, 0.62)	0.233
Patients with low C3 *n* (%)	334 (86.3)	56 (94.9)	278 (84.8)	0.039
C4 (g/L)	0.08 (0.06, 0.15)	0.07 (0.06, 0.15)	0.08 (0.06, 0.15)	0.302
Patients with low C4 *n* (%)	306 (79.1)	45 (76.3)	261 (79.6)	0.602
C-reactive protein (mg/L)	1.95 (0.84, 8.03)	5.3 (1.2, 17.85)	1.5 (0.8, 6.0)	0.002
Erythrocyte sedimentation rate (mm/h)	33 (15, 53)	37 (10, 53)	33 (16, 54)	0.776
ANA, *n* (%)	380/386 (98.4)	59/59 (100.0)	321/327 (98.2)	0.597
Anti-dsDNA, *n* (%)	331/386 (85.8)	53/59 (89.8)	278/327 (85.0)	0.420
Anti-Sm, *n* (%)	103/371 (27.8)	13/57 (22.8)	90/314 (28.7)	0.423
Anti-RNP, *n* (%)	116/371 (31.3)	19/57 (33.3)	97/314 (30.9)	0.757
Anti-SSA/Ro, *n* (%)	217/371 (58.5)	35/57 (61.4)	182/314 (58.0)	0.664
Anti-SSB/La, *n* (%)	78/371 (21.0)	12/57 (21.1)	66/314 (21.0)	1.000
Anticardiolipin-IgM, *n* (%)	53/328 (16.2)	8/48 (16.7)	45/280 (16.1)	1.000
Anticardiolipin-IgG, *n* (%)	78/328 (23.8)	13/48 (27.1)	65/280 (23.2)	0.583
SLEDAI score	16 (12, 20)	16 (13, 20)	16 (12, 20)	0.346
PASP (mmHg)	33 (29, 40)	44 (41, 52)	31 (28, 34)	NA
Pathological pattern, *n* (%)				0.196
Class I, *n* (%)	1/286 (0.3)	0/38 (0.0)	1/248 (0.4)	
Class II, *n* (%)	18/286 (6.3)	3/38 (7.9)	15/248 (6.0)	
Class III, *n* (%)	31/286 (10.8)	2/38 (5.3)	29/248 (11.7)	
Class IV, *n* (%)	151/286 (52.8)	27/38 (71.1)	124/248 (50.0)	
Class V/III + V/IV + V *n* (%)	82/286 (28.7)	6/38 (15.8)	76/248 (30.6)	
Class VI, *n* (%)	3/286 (1.0)	0/38 (0.0)	3/248 (1.2)	
Activity index	7 (5, 9)	7 (5, 10)	7 (5, 9)	0.478
Chronic index	3 (2, 4)	3 (2, 4)	3 (2, 4)	0.554

Normal distribution data were presented as average ± SD and non-normal distribution data as medians (Q25, Q75). ^a^ With PH versus without PH. ANA, anti-nuclear antibodies; anti-dsDNA, anti-double-stranded DNA; anti-Sm, anti-Smith; anti-RNP, anti-ribonucleoprotein; eGFR, estimated glomerular filtration rate; HDL-C, high-density lipoprotein cholesterol; LDL, low-density lipoprotein cholesterol; NA, not applicable; PH, pulmonary hypertension; SSA, Sjogren’s-syndrome-related antigen A; SSB, Sjogren’s-syndrome-related antigen B; SLEDAI, Systemic Lupus Erythematosus Disease Activity Index.

**Table 2:** Multivariate logistic regression analysis on risk factors of pulmonary hypertension in patients with lupus nephritis.

Variables	Odds Ratio (95% Confidence Interval)	*p* Value
Mean arterial pressure (per 1 mmHg increase)	1.05 (1.03–1.08)	<0.001
Hemoglobin (per 1 g/L increase)	0.98 (0.97–1.00)	0.014
Triglycerides (per 1 mmol/L increase)	0.71 (0.52–0.98)	0.038
C-reactive protein (per 1 mg/L increase)	1.02 (1.00–1.04)	0.088

**Table 3:** Association between pulmonary hypertension and outcomes in patients with lupus nephritis.

Variables	All-Cause Mortality		Renal Events	
	HR (95%CI)	*p* Value	HR (95%CI)	*p* Value
Unadjusted	2.46 (1.25–4.85)	0.010	2.58 (1.30–5.12)	0.007
Model 1 ^a^	2.18 (1.10–4.32)	0.025	2.49 (1.25–4.95)	0.010
Model 2 ^b^	2.01 (1.01–4.00)	0.047	2.07 (1.04–4.12)	0.039

CI, confidence interval; HR, hazard ratio. ^a^ Model 1: adjusted for age and gender. ^b^ Model 2: adjusted for Model 1 plus serum creatinine, serum albumin, and 24 h proteinuria.

## Data Availability

All data generated or analyzed during this study are included in this article. Further inquiries can be directed to the corresponding author.

## Supplementary Materials

The following supporting information can be downloaded at: https://www.mdpi.com/article/10.3390/medicina60060988/s1, Table S1: Univariate logistic regression analysis on risk factors of pulmonary hypertension in patients with lupus nephritis.
