# Supplementary material for: Prevalence, Predictors, and Outcomes of Pulmonary Hypertension in Patients with Lupus Nephritis
Source: Medicina (Kaunas). 2024 Jun 17;60(6):988. doi: 10.3390/medicina60060988 (PMC11205415; doi:10.3390/medicina60060988)
Supplement: Supplementary file 1 [file medicina-60-00988-s001.zip › medicina-2983022-20240617-supplementary.pdf]

Supplementary Table S1. Univariate logistic regression analysis on risk factors of pulmonary hypertension in patients with lupus nephritis.

Variables	Odds Ratio (95% Confidence Interval)	*p* Value
Age (per 1 year increase)	1.01 (0.99-1.03)	0.360
Female (reference to male)	0.75 (0.38-1.52)	0.430
Lupus duration on admission (per 1 month increase)	1.00 (1.00-1.01)	0.644
Lupus nephritis duration on admission (per 1 month increase)	1.00 (1.00-1.01)	0.591
Mean arterial pressure (per 1 mmHg increase)	1.04 (1.03-1.06)	<.001
Ulceration (reference to without the situation)	1.17 (0.32-4.19)	0.813
Arthritis/Myositis (reference to without the situation)	1.29 (0.15-10.92)	0.817
Pleuritis (reference to without the situation)	2.29 (1.29-4.07)	0.005
Pericarditis (reference to without the situation)	1.47 (0.78-2.77)	0.238
Vasculitis (reference to without the situation)	0.00 (0.00-Inf)	0.989
Leukocytopenia (reference to without the situation)	1.28 (0.68-2.40)	0.446
Thrombocytopenia (reference to without the situation)	0.95 (0.35-2.57)	0.927
Hemoglobin (per 1 g/L increase)	0.98 (0.96-0.99)	<.001
24 h proteinuria (per 1 g increase)	0.97 (0.89-1.05)	0.406
Blood urea nitrogen (per 1 mmol/L increase)	1.06 (1.03-1.10)	<.001
Serum creatinine (per 1 umol/L increase)	1.01 (1.01-1.01)	0.003
eGFR (per 1 mL/min/1.73 m^2 increase)	0.99 (0.98-0.99)	0.007

Serum uric acid (per 1 μmol/L increase)	1.00 (1.00-1.00)	0.149
Serum albumin (per 1 g/L increase)	1.00 (0.96-1.04)	0.863
Total cholesterol (per 1 mmol/L increase)	0.88 (0.76-1.02)	0.090
Triglycerides (per 1 mmol/L increase)	0.79 (0.62-1.01)	0.059
HDL-C (per 1 mmol/L increase)	0.62 (0.31-1.22)	0.166
LDL-C (per 1 mmol/L increase)	0.90 (0.75-1.10)	0.308
C3 (per 1 g/L increase)	0.35 (0.11-1.13)	0.080
C4 (per 1 g/L increase)	0.12 (0.00-3.44)	0.212
C-reactive protein (per 1 mg/L increase)	1.02 (1.01-1.04)	0.018
Erythrocyte sedimentation rate (per 1 mm/h increase)	1.00 (0.99-1.01)	0.921
ANA (reference to without the situation)	2876729.81 (0.00-Inf)	0.988
Anti-dsDNA (reference to without the situation)	1.56 (0.63-3.82)	0.333
Anti-Sm (reference to without the situation)	0.74 (0.38-1.43)	0.365
Anti-RNP (reference to without the situation)	1.12 (0.61-2.04)	0.715
Anti-SSA/Ro (reference to without the situation)	1.15 (0.65-2.06)	0.628
Anti-SSB/La (reference to without the situation)	1.00 (0.50-2.00)	0.995
Anticardiolipin-IgM (reference to without the situation)	1.04 (0.46-2.38)	0.918
Anticardiolipin-IgG (reference to without the situation)	1.23 (0.61-2.46)	0.561
SLEDAI score (per 1 score increase)	1.01 (0.96-1.06)	0.698

ANA, anti-nuclear antibodies; anti-dsDNA, anti-double-stranded DNA; anti-Sm, anti-Smith; anti-RNP, anti-ribonucleoprotein; eGFR, estimated glomerular filtration rate; HDL-C, high-density lipoprotein cholesterol; LDL, low-density lipoprotein cholesterol; SSA, Sjogren's-syndrome-related antigen A; SSB, Sjogren's-syndrome-related antigen B; SLEDAI, Systemic Lupus Erythematosus Disease Activity Index; Inf, Infinite.
